# Fallout of Lead Over Paris From the 2019 Notre‐Dame Cathedral Fire

**DOI:** 10.1029/2020GH000279

**Published:** 2020-08-21

**Authors:** Alexander van Geen, Yuling Yao, Tyler Ellis, Andrew Gelman

**Affiliations:** ^1^ Lamont‐Doherty Earth Observatory Columbia University Palisades NY USA; ^2^ Department of Statistics Columbia University New York, NY USA

**Keywords:** lead contamination, fallout, statistical modeling, emergency response

## Abstract

The roof and spire of Notre‐Dame cathedral in Paris that caught fire and collapsed on 15 April 2019 were covered with 460 t of lead (Pb). Government reports documented Pb deposition immediately downwind of the cathedral and a twentyfold increase in airborne Pb concentrations at a distance of 50 km in the aftermath. For this study, we collected 100 samples of surface soil from tree pits, parks, and other sites in all directions within 1 km of the cathedral. Concentrations of Pb measured by X‐ray fluorescence range from 30 to 9,000 mg/kg across the area, with a higher proportion of elevated concentrations to the northwest of the cathedral, in the direction of the wind prevailing during the fire. By integrating these observations with a Gaussian process regression model, we estimate that the average concentration of Pb in surface soil downwind of the cathedral is 430 (95% interval, 300–590) mg/kg, nearly double the average Pb concentration in the other directions of 240 (95% interval, 170–320) mg/kg. The difference corresponds to an integrated excess Pb inventory within a 1 km radius of 1.0 (95% interval, 0.5–1.5) t, about 0.2% of all the Pb covering the roof and spire. This is over 6 times the estimated amount of Pb deposited downwind 1–50 km from the cathedral. To what extent the concentrated fallout within 1 km documented here temporarily exposed the downwind population to Pb is difficult to confirm independently because too few soil, dust, and blood samples were collected immediately after the fire.

## Key Points

- Surface soil Pb concentrations within 1 km of Notre‐Dame cathedral are about 200 mg/kg higher downwind of the fire relative to background
- The corresponding fallout of 1,000 kg Pb is 6 times higher than the estimated mass of Pb from the fire transported by the wind beyond 1 km
- The resulting human exposure was probably dwarfed by the impact of leaded gasoline in previous decades but warranted more testing sooner

## 1 Introduction

**Figure 1:**
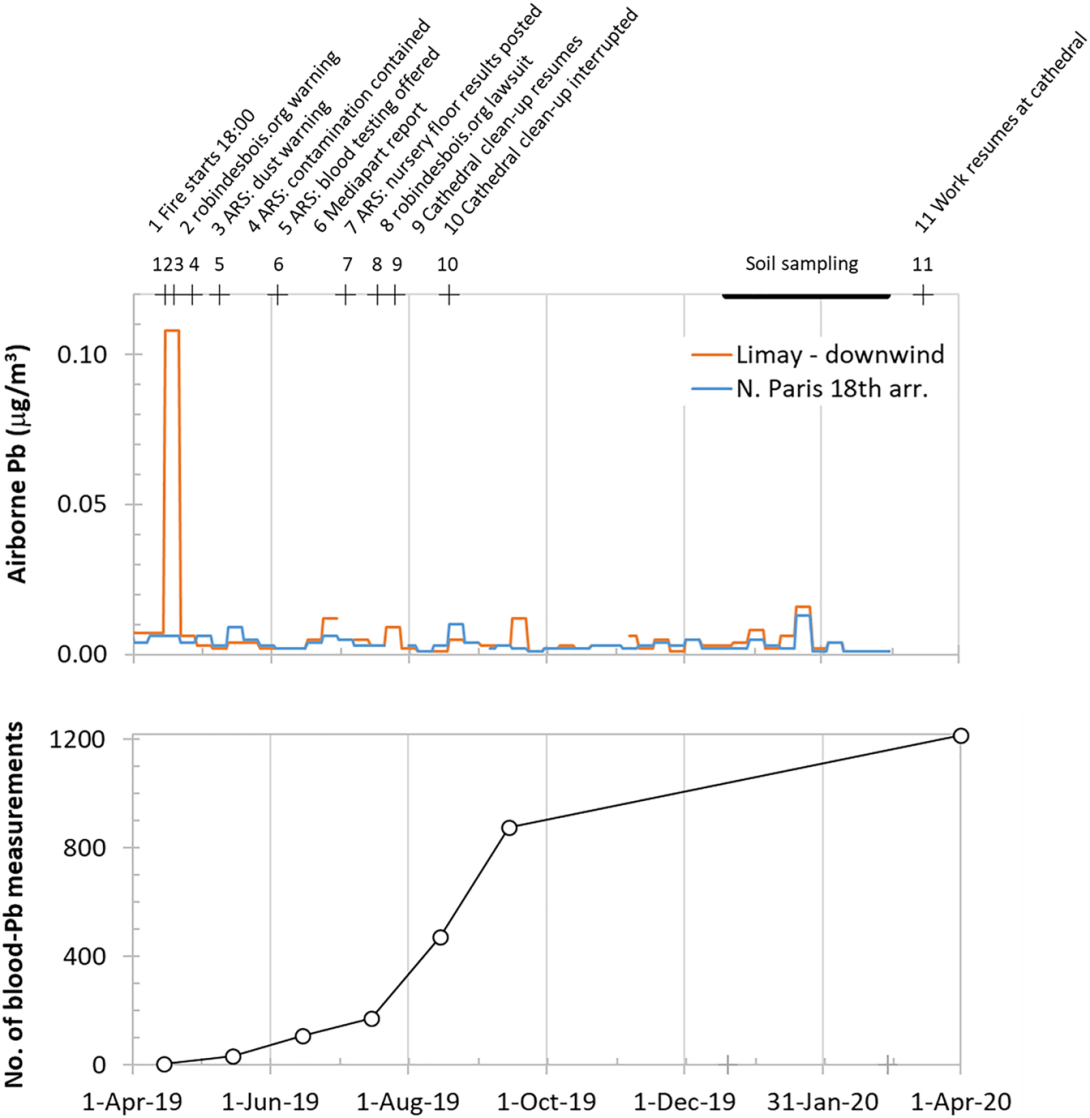
Events following the 15 April 2019 Notre‐Dame cathedral fire shown in the upper panel with weekly time series of Pb concentrations in airborne particulate matter measured at two Airparif monitoring stations (https://www.airparif.asso.fr/en/). Lower panel: total number of children and adolescents in the 1st, 4th, 5th, and 6th arrondissements whose blood was tested for Pb (Agence Régionale de la Santé, 2019g, 2019h).

The roof and spire of Notre‐Dame cathedral in the center of Paris covered with 460 t of lead (Pb) tiles burned down within a few hours of a fire that started early on the evening of 15 April 2019 and took 9 hr for the fire brigade to extinguish. The yellow color of the smoke rising from the cathedral during the first few hours has been attributed to PbO particles entrained with the hot ascending air and formed by heating to 600°C the melted Pb that accumulated on top of the vault of the cathedral (INERIS, 2019). Prevailing winds combined with modeling of the plume of smoke particles rising from the fire have linked this increase to the ejection of about 150 kg of Pb, only 0.03% of the total covering the cathedral, into the atmosphere by the fire and redeposition over several tens of kilometers. This is consistent with observations at an air quality monitoring station 50 km downwind of the burning cathedral where a twentyfold increase in particulate Pb concentration, from 0.050 to 0.105 μg/m^3^, was recorded during the week that followed the fire (Figure 1, upper panel). The same INERIS (2019) report also states that considerably more Pb was likely deposited in the immediate vicinity of the cathedral, but there was no attempt to estimate this amount.

The goal of this study was to determine if a very basic soil sampling procedure of the fallout paired with more advanced statistical analysis could yield useful information about Pb deposition resulting from the fire. Provided sampling is limited to the very surface, soil has the advantage of preserving the signal of a fallout for longer than hard surfaces such as road and sidewalks that are swept by wind and flushed by rain or have been cleaned with water. The consequences of the Notre‐Dame fire are well worth documenting because lead has neurotoxic effects even at low levels of exposure at a young age (Aizer & Currie, 2019; Lanphear et al., 2005; Laidlaw & Filippelli, 2008). Dust and soil are also known sources of child Pb exposure, including in France (ANSES, 2020; Etchevers et al., 2015; Glorennec et al., 2016).

Our surface soil data collected 9–10 months after the fire show that the population residing within 1 km and downwind of the fire was probably considerably more exposed to Pb fallout, albeit for a brief period, than indicated by measurements and surveys conducted by local authorities weeks to months later. Besides demarcating the hazard and possibly reducing exposure, more rapid collection and posting of environmental data could have avoided concerns subsequently raised about the official response to the fire and its aftermath. Other cases, albeit of a very different magnitude, where lack of data diminished public trust and led to inadequate official responses include the nuclear reactor accidents in Chernobyl and Fukushima (Alexievich, 2006; Brown et al., 2016).

## 2 Chronology and Available Data

The sequence of announcements and measures taken after the fire by local authorities provide a context for and contribute to the interpretation of the new Pb measurements presented here. Four days after the fire, on 19 April, the environmental nongovernmental organization Robin des Bois (2019) issued a press release expressing concern about the likely large quantities of Pb mobilized by the fire, referring to potential health risks incurred by firefighters, workers on the site, and the surrounding population. On 27 April, almost 2 weeks after the fire, the Agence Régionale de la Santé (2019a) co‐issued a press release indicating that dust sampling had revealed some locally elevated levels of Pb and that areas very close to the cathedral that could not rapidly be cleaned had been closed to the public. The press release also recommended that nearby inhabitants remove indoor dust with wet wipes and announced follow‐up studies to minimize risks to workers on the site and the surrounding population. On 9 May 2019, the Agence Régionale de la Santé (2019b) confirmed soil Pb levels of 10,000–20,000 mg/kg in the out‐of‐bounds area very near the cathedral but also reported that no levels above 300 mg/kg, the maximum level recommended in France (HCSP, 2014), were measured outside this area within the Île de la Cité, where the cathedral is located. The same news release from the ARS reported that no sample collected around the cathedral to assess air quality exceeded the regulatory level of 0.25 μg/m^3^ for Pb in airborne particulate matter. This indicated that Pb exposure through inhalation was unlikely, although the timing of the sampling relative to the fire was not provided.

Almost a month later, on 4 June, the Agence Régionale de la Santé (2019c) reported that indoor dust collected in some nearby apartments was found to be elevated in Pb and referred to a first tested child whose blood‐Pb content was over 50 μg/L (i.e., 5 μg/dL in the unit used in the United States), the local intervention level requiring a follow‐up investigation at home (HCSP, 2014). In the same press release, whose overall tone was meant to be reassuring, the ARS offered to test the blood of any children less than 7 years old residing on the Île de la Cité for Pb at a nearby hospital. On 18 July 2019, the Agence Régionale de la Santé (2019d) issued a 100+ page report indicating no additional blood‐Pb levels above 50 μg/L had been detected in 81 children from the 1st, 4th, 5th, and 6th arrondissements, all areas downwind of the fire, and that a Pb source unrelated to the fire was identified in the home of the previously reported child with >50 μg/L blood Pb. The same document indicated that indoor surface Pb concentrations at a number of nurseries sampled downwind of the fire were all <1,000 μg/m^2^, the local regulatory level after Pb remediation in housing, and mostly <70 μg/m^2^, the level above which a blood test is encouraged (HCSP, 2014), along with a detailed map of measurements of Pb concentrations in surface dust of the area. Unlike soil measurements, which require unconsolidated material such as a tree pit or a park, surface Pb measurements, usually conducted indoor, rely on wiping a hard surface (e.g., a floor or the top of a cabinet) over a set area with a wet tissue that is then analyzed. This is a standard regulatory procedure in France as well as in the United States (JORF, 2009; Lanphear et al., 1995).

On 26 July, Robin des Bois filed a lawsuit claiming insufficient measures were taken to protect the health of workers on the cathedral site, after which activities were interrupted for several weeks (Le Monde, 2019). Soon thereafter on 4 August, the Agence Régionale de la Santé (2019e) tried to refute allegations by Mediapart (2019), an investigative online news provider, that it was minimizing the risk of Pb exposure to the population residing downwind of the cathedral. On 27 November, however, the Agence Régionale de la Santé (2019f) announced online access to georeferenced environmental Pb data collected both before and after the cathedral fire (https://santegraphie.fr/mviewer/?config=app/notredame_od.xml). The data posted by the ARS included a dozen wipe‐based surface Pb measurements conducted in 2018 in close proximity to the cathedral and about 60 measurements of the same type in the same area from 2020. In the 2018 and 2020 data, only one measurement exceeds 5,000 μg/m^2^ Pb, and this by less than a factor of 2.

For 2019, the database contains a much larger number of measurements around the cathedral, including dozens extending over a distance of 50 km in the direction of the plume and the air quality monitoring station of Limay where an increase in airborne Pb had been detected during the week after the fire (Figure 1, upper panel). Outside a radius of 2 km from the cathedral, none of the reported measurements exceed 5,000 μg/m^2^. Between 1 and 2 km from the cathedral, a subset of 7 out of a total of ∼40 measurements, all conducted between mid‐May and mid‐June 2019, exceed 5,000 μg/m^2^ and in all but one case by less than a factor of 10. Within a radius of 1 km of the cathedral, the proportion and level of elevated surface Pb measurements is comparable to the findings in the 1–2 km range, although the majority of these measurements date from summer and fall 2019, that is, several months later. It is only within a radius of 100 m from the cathedral that much higher surface Pb concentrations, most over 100,000 μg/m^2^ and several near 1,000,000 μg/m^2^ are reported on the ARS site.

The ARS georeferenced data site only lists 24 soil Pb measurements within a radius of 2 km from the cathedral, all conducted after the fire and between April and June 2019. Most of the reported Pb concentrations are below 100 mg/kg, with 6 in the 100–300 mg/kg range, and only one higher value of 310 mg/kg within 100 m of the cathedral. These values do not seem consistent with the 10,000–20,000 mg/kg concentrations reported for the same area by the Agence Régionale de la Santé (2019b), which were not posted, unless the measurements were obtained by different methods. The soil protocol followed by the ARS calls for sampling to 5 cm depth and homogenizing this material before analysis. In the case of Pb contamination limited to the top 1 mm, this could lead to greater than fiftyfold lower concentrations than measured from the very surface with a handheld X‐ray fluorescence (XRF) analyzer (Landes et al., 2019). Diluting the highest reported surface Pb concentration of 1,000,000 μg/m^2^ over the mass of soil to 5 cm depth would, for instance, increase the soil Pb concentration by only 10 mg/kg, that is, little more than 10% of background levels based on the other measurements. The relatively low soil concentrations posted on the ARS site are therefore not necessarily inconsistent with the much higher levels referred to in the earlier press release.

## 3 Materials and Methods

### 3.1 Data Collection

**Figure 2:**
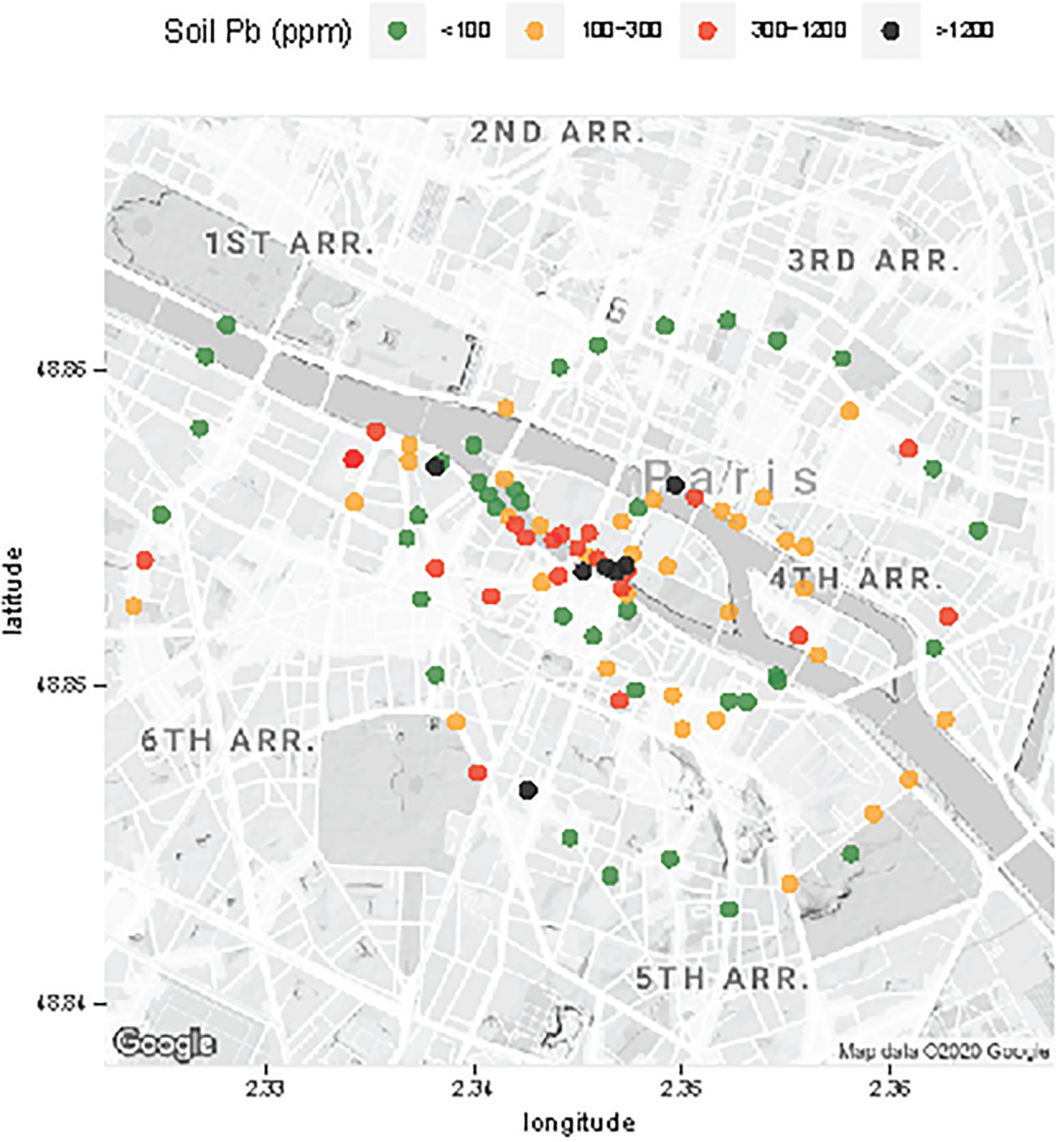
Map of 100 soil sample locations around the cathedral and their Pb concentrations. The two circles of samples centered on the cathedral have radii of 400 and 1,000 m, respectively. Additional samples were collected in downwind direction, northwest of the cathedral.

One hundred soil samples were collected between 20 December 2019 and 29 February 2020 mostly from tree pits (55 samples) and parks or smaller garden‐like areas (30). In a few cases, samples were collected from small gaps in the sidewalk (13) or even semipermanent plant pots (2) for lack of more suitable alternatives. One set of 58 soil samples were spaced roughly equally along two concentric circles of 400 and 1,000 m in radius centered on the cathedral (Figure 2). The remaining 42 samples targeted the area likely to have been impacted by fallout from fire, downwind of the cathedral.

A large metal spoon was used to recover ∼50 g of material from the upper ∼1 cm of each site. The samples were air‐dried overnight in paper bags, after which the fine fraction was separated through a metal kitchen sieve (∼1 mm mesh size) and poured into 20 mL scintillation vials. Without further processing, the fine fraction was analyzed in the inverted vials through plastic cling wrap using a handheld Innov‐X (now Olympus) Delta Premium XRF analyzer. The XRF's internal calibration was confirmed by bookending both rounds of analyses with Standard Reference Material soil 2711a from the U.S. National Institute of Standards and Technology. The average of 1,480±40 mg/kg (*n*=4) obtained for Pb was consistent with the certified value of 1,400 ± 10 mg/kg, and the data are therefore reported without further adjustment.

The XRF measures the concentrations of 16 additional elements. Tin (Sn) is of particular interest for the present study but there is no certified Sn value for SRM 2711a. Landes (2019) compared soil Sn concentrations measured by the same instrument with two dozen soil digests analyzed by inductively coupled plasma mass spectrometry (Cheng et al., 2004). The slope of Sn concentrations measured by XRF as a function of concentrations measured by ICPMS of 1.6 indicates a systematic overestimate of Sn concentrations by XRF.

Besides a map, soil Pb concentrations are also displayed in a polar coordinate system centered on the cathedral to help to visualize the impact of the fire independently of the presumed direction of the plume. The sampled Pb peaks at the northwest, and as a whole, drops off with a longer radial distance, while the slope inside the plume is sharper. Based on INERIS (2019), we specify the plume region to be the sector between 260° to 310° clockwise from the cathedral independently from the Pb data.

### 3.2 Notation and Preprocessing

We denote the soil Pb concentration (in mg/kg) in the *i*th location to be *y*_*i*_, *i*=1,…,*n*, and compute the its radical distance *r*_*i*_ (in km) and the bearing *θ*_*i*_ (in degrees, North = 0, East = 90) from the cathedral. We index the type of soil by *k*[*i*]∈{1,2,…,5} to represent where the *i*th sample was drawn from: cracks in the sidewalk, smaller garden areas, park, plant pots, or tree pits.

As for many other natural measurements, the observed *y*_*i*_ has a heavy right tail. Directly modeling *y* will cause the model to be overly sensitive to a few extreme values. Measurement errors in chemical analysis are often additive in the lower end and multiplicative in the high end. Instead of a log transformation, we therefore select a 1/4th power transformation, as the measurement errors would likely be of similar order of magnitude in different sites. For notation simplicity, we substitute *y* ^1/4^→*y* in the model description we use and transform it back to the ordinary scale after model fitting.

The concentration of soil Pb varies both spatially and by soil type. We decompose the outcome *y*_*i*_ into three terms:

$$y_{i}=\mu_{k[i]}+f(r_{i},\theta_{i})+\epsilon_{i},\;\epsilon_{i}∼N(0,\sigma^{2}),\;i=1,\ldots ,n,$$

which includes

- the soil type coefficient *μ*_*k*_[*i*], which depends only on what type of soil the sample belongs to;
- the spatial term *f* (*r*_*i*_,*θ*_*i*_), which depends only on where the sample is collected (encoded by distance and bearing);
- and an independent observational noise *ϵ*_*i*_, which contains measurement error, small‐scale fluctuations, and effects from any unmeasured covariates.

### 3.3 Hierarchical Modeling of Different Soil Types

**Figure 3:**
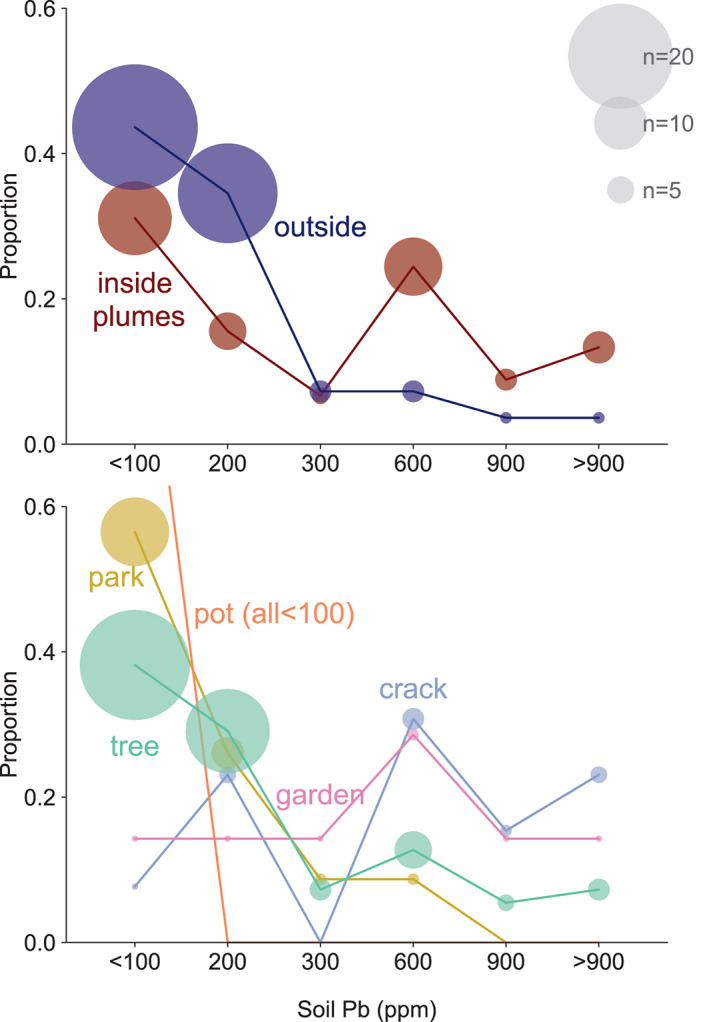
Upper panel: Proportion of soil Pb collected inside and outside the area passed over by the plume of smoke rising from the cathedral. Lower panel: Proportion of Pb sample for different types of soils. The size of the symbols indicates the number of samples in each grouping. The two plant pots are low in Pb and their symbol out of range.

**Figure 4:**
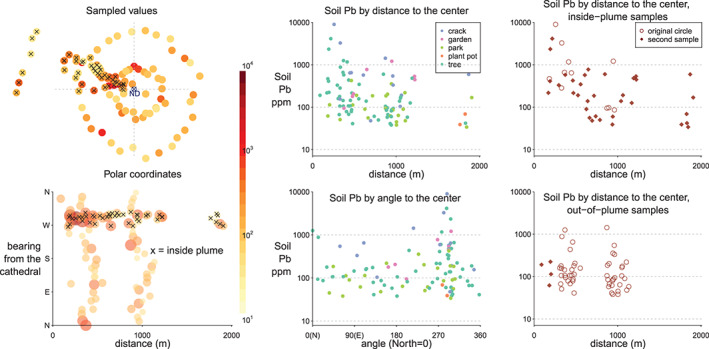
Left column: sampled locations and Pb concentrations in both Cartesian and polar coordinates. Middle column: scatter plot of soil Pb by distance and bearing from the cathedral, colored by soil type. Right column: soil Pb by distance from the cathedral, grouped by inside/outside plume.

The lower panel of Figure 3 and the middle column of Figure 4 suggest that the type of soil (tree pit, park, smaller garden areas, cracks in the sidewalk, and plant pots) is predictive of Pb concentrations. The sample sizes in different types are unbalanced, and a simple sample mean is noisy for groups with small samples. To partially pool across the data, we fit a hierarchical model to the soil type coefficients *μ*_*k*_ (Gelman & Hill, 2006).

However the model is not identifiable yet, as an additive constant can be extracted from the *μ* and added to *f*. To resolve this, we restrict the soil type coefficients by a zero‐sum constraint, $\sum_{k=1}^{5}\mu_{k}=0$.

### 3.4 Modeling the Pb Distribution by a Gaussian Process Regression

We model the spatial pattern nonparametrically by placing a mean‐zero Gaussian process prior on the latent function *f*. It models the joint distribution at any two locations, *f* (*r*,*θ*), *f* (*r* *′*,*θ* *′*), using a multivariate Gaussian distribution. To flexibly account for the influence from the distance, bearing, and their interactions, we use a product kernel in the Gaussian process prior:

$$K(r_{1},\theta_{1},r_{2},\theta_{2}):=\text{Cov}(\;f(r_{1},\theta_{1}),f(r_{2},\theta_{2}))=\alpha K_{1}(r_{1},r_{2})K_{2}(\theta_{1},\theta_{2}),$$

where for distances, we adopt the commonly used squared exponential kernel:

$$K_{1}(r_{1},r_{2})=\exp\left(-\frac{{\left(r_{1}-r_{2}\right)}^{2}}{\rho_{r}^{2}}\right).$$

For the bearing, we employ a periodic kernel:

$$K_{2}(\theta_{1},\theta_{2})=\exp\left(-\frac{2\sin^{2}(\pi |\theta_{1}-\theta_{2}|/360)}{\rho_{\theta }^{2}}\right).$$

Besides the soil type effect *μ*, spatial latent function *f*, and scale of the observational variation *σ*, the model also contains hyperparameters *α*: the scale of the spatial signal (how strong the spatial pattern is); *ρ*_*d*_: the length scale in the distance dimension (how rigid the function *f* can change over distance); and *ρ*_*θ*_: the length scale in the angle dimension.

Since the modeled outcome *y* ^1/4^ and the distance (in km) are all roughly unit scaled, we adopt weakly informative priors

$$\rho_{d}∼N(0,1.5^{2}),\;\rho_{\theta }∼N(0,1),\;\alpha ,\sigma ∼N(0,6^{2}),\;\mu_{k}∼N(0,1),\;k=1,\ldots ,5.$$

We sample from the posterior distribution of all parameters in the model using Stan (Stan Development Team, 2020). In our example, the chains mixed well for 4 chains and 3,000 iterations per chain.

### 3.5 Inference From the Fitted Model

We sample a uniform 30×30 grid of locations $\left(\tilde{\rho },\tilde{\theta }\right)$ in the 1.5 km neighborhood. After integrating out the posterior distribution, we obtain the posterior predictive distribution of the outcome values at this location $\tilde{f}=f(\tilde{\rho },\tilde{\theta })$ is from

(1) $$\tilde{f}|\tilde{\rho },\tilde{\theta },\rho ,\theta ,f∼N\left(K(\tilde{\rho },\tilde{\theta },\rho ,\theta )K^{-1}(\rho ,\theta )f,K(\tilde{\rho },\tilde{\theta })-K(\tilde{\rho },\tilde{\theta })K^{-1}(\rho ,\theta )K(\rho ,\theta ,\tilde{\rho },\tilde{\theta })\right).$$

We model the outcome to the 1/4 power and transform *f* back to *f* ^4^ in the visualizations.

Further, we add the observational noise and generate the posterior predictive distribution of $\tilde{y}$ outcome $\tilde{y}$ in location $\left(\tilde{\rho },\tilde{\theta }\right)$ by location $\tilde{f}=f(\tilde{\rho },\tilde{\theta })$ is from

(2) $$\tilde{y}|\tilde{\;f}∼N(\tilde{\;f}|\sigma_{\text{sim}}^{2}),\;\sigma_{\text{sim}}∼p(\sigma |y).$$

This amounts to the prediction of the outcome in a typical soil type with *μ*=0 such that we can make fair comparison of pure spatial effects in the later sections.

We do not impute locations with $\tilde{r}<100$ m. We do not have any data in that region and any inference relies on extrapolation.

The plume is a sector defined by C={θ: 260°< *θ* < 310°}. At each distance $\tilde{r}$, we compute the plume excess, the difference of soil Pb (ppm) between the plumes on the outside along the any a ring with any given radius. We further aggregate the excess amount of Pb in the plume within any circle (see Supporting Information).

## 4 Results

### 4.1 Raw Data Summary

Concentrations of Pb measured in all surface soil samples range from 30 to 9,000 mg/kg and average 400 mg/kg (median of 140 mg/kg). All four Pb concentrations >2,000 mg/kg are inside the plume area and within a distance of 400 m from the cathedral. Overall, soil Pb concentrations average 200 mg/kg outside (*n*=45) and 400 mg/kg (*n*=55) inside the plume area, respectively (Figure 2). Average soil Pb for tree pits (300 mg/kg; *n*=55) and garden areas (500 mg/kg; *n*=7) are comparable, but markedly lower in park areas (130 mg/kg; *n*=23). Cracks in the sidewalk (1,400 mg/kg, *n*=13) on the other hand are often higher in Pb than neighboring tree pits and garden areas (Figure 3). Among the other soil constituents analyzed by XRF, only Sn shows a systematic relationship with Pb, and this at the higher concentrations. For eight samples in the 1,000–9,000 mg/kg range of Pb concentrations, the mass ratio of Sn relative to Pb averages 3.5% after recalibrating the XRF signal.

Unlike air, water, and food, there is no standard in France for the Pb content of soil in outdoor public areas. A recommendation from French health authorities of 300 mg/kg corresponds approximately to the level at which blood‐Pb of 5% of infants coming in contact with the soil would exceed a threshold of 50 μg/L (HCSP, 2014). For comparison, the current U.S. Environmental Protection Agency standard for residential soil in areas where children play is 400 mg/kg Pb, but lowering this value is under discussion. Relative to 300 mg/kg, the Pb content of 29 of our 100 samples exceeds the French recommended value, 21 of which inside the plume area and 8 outside (Figure 3). Considering only the samples collected along the two concentric circles to avoid bias, the average soil Pb content within the plume is 500 ± 200 mg/kg (*n*=7, 1 sd), compared to 200 ± 40 mg/kg (*n*=51) outside the plume (Figure 4).

### 4.2 Model Inference

**Figure 5:**
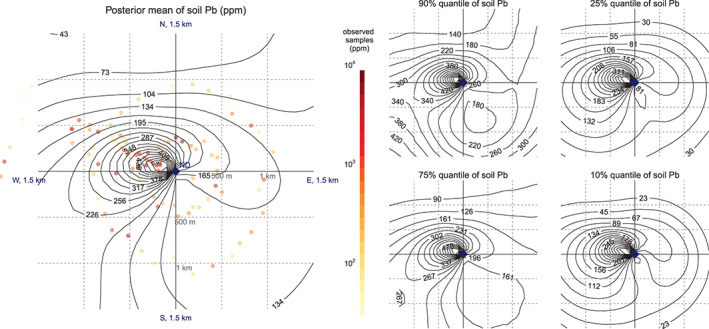
Contour plot of posterior mean and quantiles of $\tilde{f}$ (net of soil types and measurement errors) of soil Pb concentrations within 1.5 km of the cathedral.

Contours of modeled Pb concentrations also show more elevated levels in a northwesterly direction from the cathedral compared to other areas (Figure 5). Although this peak was identified independently, it corresponds closely to the direction of the plume derived from meteorological observations INERIS (2019). Bayesian inference encodes all uncertainty, which is displayed as 90%, 75%, 25%, and 10% quantiles of the predicted spatial concentration of soil Pb concentrations $f(\tilde{r},\tilde{\theta })$, at all imputed locations among the 1.5 km neighborhood around the cathedral (Figure 5). The estimation separates all measurement errors and soil types. In locations where less data were collected, south and east of the cathedral, the model essentially has to extrapolate and the posterior standard deviation of $\tilde{f}$ is consequently large.

**Figure 6:**
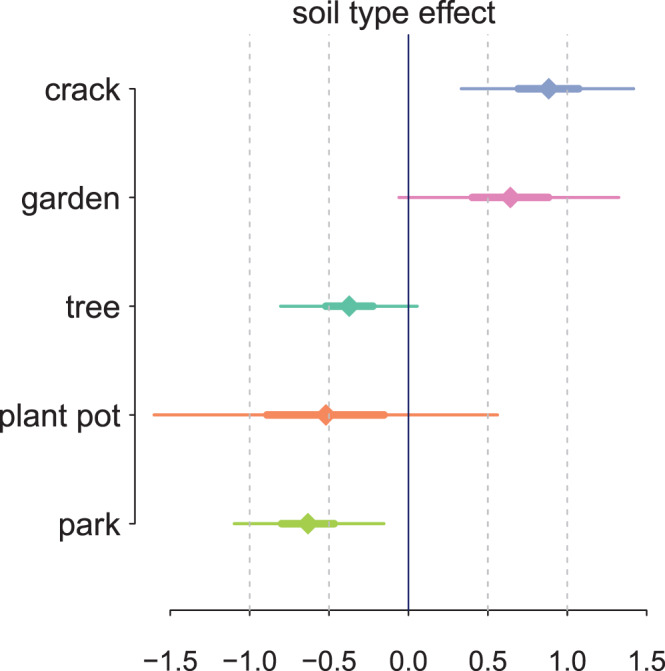
Posterior mean and 95% intervals for soil type effects *μ*_*k*_.

The effect of soil type indicates a decline in Pb concentrations from cracks in the sidewalk to tree pits and parks (Figure 6). The effect of areas described as gardens is more variable, and poorly constrained in the two cases of the two plant pots. The coefficient is on the *y* ^1/4^ scale; for a median value *y*≈140, an additive effect of 0.5 (−0.5) on *y* ^1/4^ corresponds to 100 (−65) mg/kg increase on *y*.

**Figure 7:**
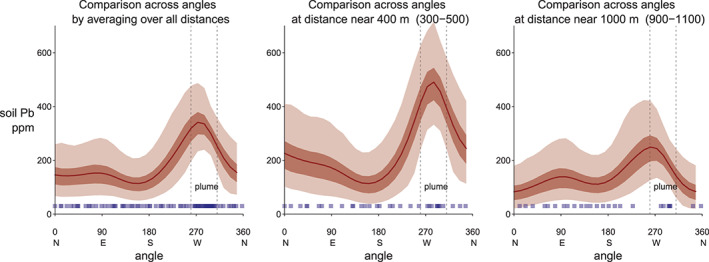
Modeled Pb concentrations as a function of direction in relation to the cathedral, evaluated at distances of 400 and 1,000 m and averaged over all distances <1.5 km.

The model estimates Pb concentrations *f* as a function of the bearing from the cathedral, evaluated at distances of 400 and 1,000 m and average concentrations over all distances <1.5 km (Figure 7). At the 400 m ring, the soil Pb for outside‐plume‐average is about 190 (95% interval, 130–270) mg/kg and peaks at 490 (95% interval, 330–710) mg/kg in the core of the plume.

**Figure 8:**
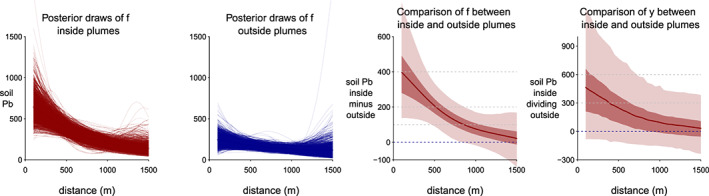
From left, first and second panels: Posterior draws of mean Pb concentrations $\tilde{f}$ inside and outside plumes as a function of distance from the cathedral. The uncertainty increases where there is little data. Third panel: The posterior mean of the difference between and inside and outside the plume (Supporting Information Equation 3). Fourth panel: Comparison of predicted to observed $\tilde{y}$ Pb concentrations (Supporting Information Equation 4). With additional observational noise added, the uncertainty interval is much wider.

The posterior predictive distribution of ${\text{Excess}}^{\;f}(\tilde{r})$ (Supporting Information Equation 3) shows that the difference in Pb concentration between inside and outside the plume declines from 350 (95% interval, 140–640) mg/kg at 200 m from the cathedral to 200 (95% interval, 90–330) mg/kg at 500 m, and 90 (95% interval, 0–190) mg/kg at 900 m, respectively, and vanishes beyond that distance (Figure 8).

**Figure 9:**
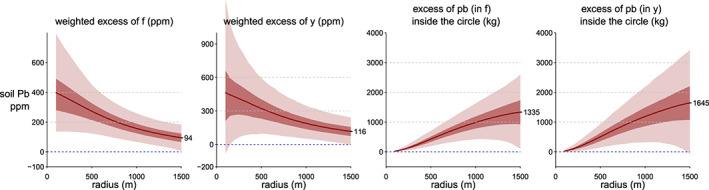
From left, first and second panels: Average excess Pb (Supporting Information Equations 5–6) inside circle of radius *r* for the mean response *f* and observation *y*. Third and fourth panels: Accumulated excess Pb (Supporting Information Equation 7) inside circle of radius *r*.

The model also calculates the average excess Pb inside a given radius (see Supporting Information) on both the mean response *f* and with additional observational noise respectively (Figure 9). On the observational level *y*, inside the 1 km circle, the average concentration of Pb inside the plume is 430 (95% interval, 300–590) mg/kg and nearly double the average Pb concentration in the other directions of 240 (95% interval, 170–320) mg/kg. Finally, the model calculates the corresponding integrated mass of excess is 1,000 kg (95% interval, 500–1,500) kg of Pb at a 1,000 m distance from the cathedral and becomes poorly constrained beyond that distance for lack of data (Figure 9).

## 5 Discussion

Soil Pb concentrations around Notre‐Dame cathedral show considerable spatial variability, both inside and outside the plume area. In some cases, this may reflect site‐specific factors such as newly added soil (Figure 6). This may be why park areas are generally lower in Pb. Cracks in the sidewalk, on the other hand, are generally higher in Pb possibly because of a preserved legacy of contamination from decades of leaded gasoline use. An occasional highly local source of contamination from Pb paint or other sources cannot be ruled out, although these were evidently not sufficient to erase a pattern that is consistent with the trajectory of the plume. The model effectively subtracts systematic differences in background Pb concentrations for different soil types when calculating the excess inside the plume to outside the plume.

The Pb tiles covering the roof of the cathedral and the spire date to the second half of the nineteenth century (Daly, 1866). Some combination of Sn and Pb in solder was probably used extensively to cover the roof and spire of the cathedral. The constant proportion of Sn relatively to Pb in the soil with high levels of Pb can therefore be attributed to the fire. Concentrations of Sn relative to Pb are not sufficiently elevated, however, to separate different sources of Pb at lower levels of contamination.

The background level below 200 mg/kg Pb outside the plume is plausible given local background levels of 20 mg/kg with, in addition, a legacy of leaded gasoline use until 2000 (Miquel, 2001; Saby et al., 2006). Without the model, the difference in Pb concentrations between the area inside and outside the plume would have been poorly constrained (Figure 8). A key question is the extent to which this excess is representative of the overall fallout over the plume area, including hard surfaces such as sidewalks and roads where this excess could have been washed away. Only 3 mm of rain was recorded during the week following the fire, but a total of 92 mm fell over Paris within 4 weeks of the fire (https://www.historique-meteo.net/france/ile-de-france/paris/2019/04/).

Lead has a particularly strong tendency to adsorb to mineral surfaces (Selim, 2017). Once in contact with soil, Pb is therefore unlikely to be flushed off of particles by water, especially within less than a year, unless by physical removal of the soil. If anything, tree pits might be concentrating Pb from a larger area if surface runoff percolates through tree pits and supplies particles from nearby hard surfaces. However, this also seems unlikely given the extensive drainage system along the sides of the streets of Paris, which is always lower in elevation than the sampling sites.

A more likely mechanism for concentrating Pb in tree pits is capture of airborne particles by tree leaves, followed by rainfall rinsing the leaves or settling of the leaves into the tree pit. Studies of the natural radioisotope ^210^Pb, whose atmospheric fallout is known, have shown that this process can enhance its accumulation by one thirds to two thirds under the canopy of trees (Fowler et al., 2004), but not by an order of magnitude. Parks without trees, on the other hand, should not be subject to this process and might be more indicative of the fallout, at least in the short term and before erosion or the addition of new soil.

For comparison of our estimate of 1,000 kg of excess Pb deposited downwind of the fire, the 50 km long plume emanating from fire beyond a distance of 1 km was estimated to contain about 150 kg Pb on the basis of a furnace experiment using a combination metallic Pb and plastic INERIS (2019). Whereas the possibility of preferential accumulation of Pb in tree pits cannot be ruled out, the amount of Pb deposited within 1,000 m of the cathedral estimated from the soil survey is fairly well constrained. About 6 times more Pb was therefore deposited within 100–1,000 m of the cathedral than beyond that distance. For perspective, the addition of Pb to gasoline resulted in air emission of 4,100 t of Pb per year in France in 1990 (Miquel, 2001). Using population as a proxy for traffic and accounting for the one‐fifth proportion of the French population residing in the greater Paris region, this suggests that the population of the city was exposed at the time to emissions of about 800 t of Pb every year. Leaded gasoline was banned in 2000, and airborne emissions of Pb have dropped by at least an order of magnitude since (Motelay‐Massei et al., 2005). The impact of the Notre‐Dame fire would therefore have been dwarfed by the impact of automobile traffic a few decades ago and would have been much harder to detect in soil at the time.

A puzzle arises when the average excess of 200 ppm/kg Pb in the plume is converted, using our approximate sampling depth of 1 cm, to 4,000,000 μg/m^2^ Pb, the unit and type of measurement more frequently referred to in regulation of indoor surfaces, including in schools. Such very high levels are reported on the interactive ARS map only within 100 m of the cathedral itself, in an area that was still out of bounds for the general public as of May 2020. At greater distances, but still within 1,000 m of the cathedral, reported values are all below 20,000 μg/m^2^. Many of the reported measurements, however, date from summer 2019 or later, by which time much of the Pb fallout could have been flushed off hard surfaces such sidewalks and roadways by rain or washing. Even if our soil Pb measurements could overestimate the overall Pb fallout by a factor of 2 because of local concentration, it appears likely that the measurements based on outdoor surface wipes reported by the government considerably underestimate the amount of the Pb that was actually deposited in the plume area because of their timing. Concentrations of Pb on hard surfaces are likely to return more rapidly to background than in soil, whose retained inventory therefore provides a better record of the fallout from the fire.

What are the implications of the soil‐based findings for human exposure in the plume area in the aftermath of the fire, especially for small children who are most vulnerable? Children are not likely to play around the tree pits themselves or even the sampling sites designated as gardens, many of which are not suitable playing areas (see interactive map with photos listed under the Data Availability section). Fortunately, the more likely playing areas such as parks were generally low in Pb (Figures 4 and 6). The potential source of exposure therefore lies elsewhere and would have been the dust deposited during and immediately after the fire. This impact is difficult to ascertain from public sources for lack of specific information about in‐house swipe measurements and a sufficient number of timely blood Pb measurements (Figure 1). Unlike in New York City, for instance, infants are not systematically tested for blood Pb in France and their exposure before the fire is therefore also not well known.

Seven weeks after the cathedral fire, local authorities offered to test children from volunteer families, but the number of tests remained very low through July 2019. After exposure ends, blood‐Pb levels can decline within a few weeks although it can also take much longer (Barbosa et al., 2005). The low proportion (1%) of children reported with blood‐Pb levels >50 μg/L is welcome news but may mask a temporarily much higher level of exposure in the days to a few weeks after the fire. The few cases of surfaces elevated in Pb reported for schools in the affected area also date from summer 2019 and therefore likely underestimate peak exposure in the 1–2 weeks following the fire, especially if the schools had followed earlier recommendations and already cleaned the common areas. Finally, because the blood survey was relying on volunteers instead of proactively seeking all 6,000 potentially exposed children in the affected area through a door‐to‐door survey, it was probably biased toward a more educated, wealthier segment of the population that may have been less at risk. In a postcoronavirus world, the need and feasibility of a testing campaign of the magnitude commensurate with the scale of a large fire or other environmental accident has become much harder to argue against.

## 6 Conclusions

A report issued by the Agence Régionale de la Santé (2019h) on 16 April 2020, exactly 1 year after the fire, acknowledges the possibility that more people than indicated by the available data may have been exposed to Pb as a result of the cathedral fire. Our observations support this scenario by showing that an excess of 200 mg/kg Pb in surface soil within 1 km and downwind of the cathedral corresponds to levels of contamination previously reported only within 100 m of the cathedral during summer 2019, several months after the fire. Therefore, elevated levels of Pb in indoor dust probably extended up to 1 km from the cathedral as well.

From a disaster response perspective, our findings show that the administration of large cities such as Paris should have a large environmental investigation team on standby, ready to be deployed to make hundreds of measurements immediately after an accident or toxic spill that could potentially pose a threat to public health. There is such a team operating at the regional and national level (https://laboratoirecentral.interieur.gouv.fr/Presentation/Le-LCPP/Panorama), which was deployed and collected Pb data after the fire, but apparently not soon enough and not at the required scale. The results from this investigation could also have been communicated considerably sooner in ways that allow the public to know exactly where the hazards are, which is easy today using the mapping function of smartphones. Finally, local public health authorities could have collected environmental and biomarker data by going door to door to all families with children at risk instead of waiting for volunteers.

## Conflict of Interest

The authors declare no conflicts of interest relevant to this study.

## Supporting information



Supporting Information S1

## Data Availability

The entire data set and an interactive map of the test results with photos of each sampling site are available at https://shorturl.at/kuvD5 and https://blogs.ei.columbia.edu/wp-content/data-viz/notre-dame-lead-map/ websites, and the replication R and Stan code at https://github.com/yao-yl/parisPb website. The data are also available online (https://www.essoar.org/doi/abs/10.1002/essoar.10503270.2).
