# Supplementary material for: Fallout of Lead Over Paris From the 2019 Notre‐Dame Cathedral Fire
Source: Geohealth. 2020 Aug 21;4(8):e2020GH000279. doi: 10.1029/2020GH000279 (PMC8027784; doi:10.1029/2020GH000279)
Supplement: Supplementary file 1 — Supporting Information S1 [file GH2-4-e2020GH000279-s001.pdf]

GeoHealth

Supporting Information for

Fallout of Lead over Paris from the 2019 Notre-Dame Cathedral Fire

Alexander van Geen[1], Yuling Yao[2], Tyler Ellis[1], and Andrew Gelman[2]

[1]Lamont-Doherty Earth Observatory of Columbia University. Palisades, NY 10964, USA

[2]Department of Statistics, Columbia University. New York, NY 10027, USA

Contents of this file

Derivations for calculating excess Pb concentrations and inventories.

Excess Pb along a ring. The plume is a sector defined by $\mathcal{C} = \{\theta : 260^\circ < \theta < 310^\circ\}$. At each distance $\tilde{r}$, we compute the plume excess, the difference of soil Pb (ppm) between the plumes on the outside along the radius r ring, by

$$\text{Excess}^f(\tilde{r}) = \frac{\sum_{j:\tilde{\theta}_j \in \mathcal{C}} f(\tilde{\theta}_j, \tilde{r})}{\sum_{j:\theta_j \in \mathcal{C}} 1} - \frac{\sum_{j:\tilde{\theta}_j \notin \mathcal{C}} f(\tilde{\theta}_j, \tilde{r})}{\sum_{j:\theta_j \notin \mathcal{C}} 1}, \tag{3}$$

where $f(\tilde{\theta}_j, \tilde{r})$ is a posterior predictive draw of $\tilde{f}$ at location $\tilde{\theta}_j, \tilde{r}$ using (1). Because it is calculated using posterior draws, expression (3) is a random variable whose posterior distribution we can summarize using the mean or quantile of its simulation draws.

Likewise we compute the excess Pb at the observational level,

$$\text{Excess}^y(\tilde{r}) = \frac{\sum_{j:\tilde{\theta}_j \in \mathcal{C}} y(\tilde{\theta}_j, \tilde{r})}{\sum_{j:\theta_j \in \mathcal{C}} 1} - \frac{\sum_{j:\tilde{\theta}_j \notin \mathcal{C}} y(\tilde{\theta}_j, \tilde{r})}{\sum_{j:\theta_j \notin \mathcal{C}} 1}. \tag{4}$$

where $y(\tilde{\theta}_j, \tilde{r})$ denotes a posterior predictive draw of $\tilde{y}|\tilde{f}(\tilde{\theta}_j, \tilde{r})$ using (2). In general, $\text{Excess}^y(\tilde{r})$ has a lager posterior variance. It is because of the noise ϵ: such that there are more uncertainty even if we repeat sampling in the same location. It also has larger posterior mean than $\text{Excess}^f(\tilde{r})$. This is due to the multiplicative measurement error in ϵ. For example, if there is a multiplicative noise source that will halve or double the true value f with equal probability, the posterior mean of y becomes $\frac{2+0.5}{2} f = 1.25f$.

Excess Pb inside a circle. We further aggregate the the excess amount of Pb in the plume within the circle of radius r. To this end, we reweigh the excess density of the ring by its radius,

$$\text{Excess}^f_{\text{circle}}(\tilde{r}) = \int_0^{\tilde{r}} r' \text{Excess}^f(r') dr', \tag{5}$$

and

$$\text{Excess}^y_{\text{circle}}(\tilde{r}) = \int_0^{\tilde{r}} r' \text{Excess}^y(r') dr'. \tag{6}$$

Finally we estimate the excess amount of Pb of the plume inside a circle with any given any radius $\tilde{r}$ by

$$\frac{|\mathcal{C}|}{360} \times \pi \tilde{r}^2 \times \text{thickness} \times \text{soil density} \times \text{Excess}^y_{\text{circle}}(\tilde{r}). \tag{7}$$

For all the above quantities we compute the posterior mean, 50%, and 95% intervals using the simulation draws, a soil thickness of 1 cm, and a dry bulk soil density of 1.3 g/cm3. Differences in soil density between sampling sites are not considered.
